# Continuous electroencephalography in the surgical ICU

**DOI:** 10.1186/cc9752

**Published:** 2011-03-11

**Authors:** A Wahl, P Kurtz, R Bauer, L Hirsch, J Claassen

**Affiliations:** 1 Columbia University Medical Center, New York, USA; 2 Casa de Saúde São José, Rio de Janeiro, Brazil

## Introduction

The objective of this study is to investigate the prevalence, risk factors, and impact on outcome of electrographic seizures (ESz), nonconvulsive status epilepticus (NCSE), and periodic epileptiform discharges (PEDs) in surgical ICU (SICU) patients.

## Methods

This was a retrospective study of 156 consecutive SICU patients (mean age 65 years old (IQR 54 to 74); 40% women) who underwent continuous electroencephalography (cEEG) monitoring for altered mental status. Poor outcome was defined as death or severe disability (Glasgow Outcome Score 4 or 5).

## Results

The majority of patients were admitted following abdominal surgery (36%) and post liver transplant (24%). Sepsis developed in 102 (65%) patients, almost all patients were mechanically ventilated (94%) and approximately one-half were comatose at the time of EEG monitoring (55%). Sixteen percent (*n *= 25) had ESz, 5% (*n *= 8) NCSE, and 29% (*n *= 45) had PEDs. All eight patients with NCSE were septic. Comatose patients and those with previous liver disease were more likely to have ESz or PEDs compared with noncomatose and those with normal liver function (42% vs. 19%; *P *= 0.002 and 25% vs. 9%; *P *= 0.007, respectively). After controlling for age, coma, and organ dysfunction, the presence of ESz was independently associated with death at hospital discharge (75% with vs. 43% without ESz; adjusted OR = 3.4 (95% CI = 1.04 to 10.9); *P *= 0.04).

## Conclusions

In patients admitted to the SICU, ESz and PEDs are frequent and associated with poor outcome.

